# Synergistic Effect, Improved Cell Selectivity, and Elucidating the Action Mechanism of Antimicrobial Peptide YS12

**DOI:** 10.3390/ijms241713522

**Published:** 2023-08-31

**Authors:** Suzia Aktar Suchi, Dae Young Lee, Young Kyun Kim, Seong Soo Kang, Tahmina Bilkis, Jin Cheol Yoo

**Affiliations:** 1 Department of Pharmacy, College of Pharmacy, Chosun University, Gwangju 61452, Republic of Korea; 2 Department of Herbal Crop Research, National Institute of Horticultural and Herbal Science, RDA, Eumseong 27709, Republic of Korea; 3 Department of Veterinary Medicine and BK21 Four Program, Chonnam National University, Gwangju 61186, Republic of Korea; 4 Department of Biomedical Sciences, Chosun University, Gwangju 61452, Republic of Korea

**Keywords:** antimicrobial peptides (AMPs), fractional inhibitory concentration index (FICI), cell selectivity, mechanism of action

## Abstract

Antimicrobial peptides (AMPs) have attracted considerable attention as potential substitutes for traditional antibiotics. In our previous research, a novel antimicrobial peptide YS12 derived from the *Bacillus velezensis* strain showed broad-spectrum antimicrobial activity against Gram-positive and Gram-negative bacteria. In this study, the fractional inhibitory concentration index (FICI) indicated that combining YS12 with commercial antibiotics produced a synergistic effect. Following these findings, the combination of YS12 with an antibiotic resulted in a faster killing effect against bacterial strains compared to the treatment with the peptide YS12 or antibiotic alone. The peptide YS12 maintained its antimicrobial activity under different physiological salts (Na^+^, Mg^2+^, and Fe^3+^). Most importantly, YS12 exhibited no cytotoxicity towards Raw 264.7 cells and showed low hemolytic activity, whereas positive control melittin indicated extremely high toxicity. In terms of mode of action, we found that peptide YS12 was able to bind with LPS through electrostatic interaction. The results from fluorescent measurement revealed that peptide YS12 damaged the integrity of the bacterial membrane. Confocal laser microscopy further confirmed that the localization of peptide YS12 was almost in the cytoplasm of the cells. Peptide YS12 also exhibited anti-inflammatory activity by reducing the release of LPS-induced pro-inflammatory mediators such as TNF-α, IL-1β, and NO. Collectively, these properties strongly suggest that the antimicrobial peptide YS12 may be a promising candidate for treating microbial infections and inflammation.

## 1. Introduction

Nowadays, the widespread use of antibiotics causes the emergence of drug-resistant bacteria, which is now a major public health threat [1,2]. Different pathogenic microorganisms emerge quickly, and the rate of development of new antibiotics is slower, which ultimately results in antibiotic resistance [3]. The scientific world has devoted a great effort to find new, safer, and more effective antimicrobial agents from natural sources including plants, animals, microorganisms, etc. [4,5]. One of the most prominent classes of antibacterial agents from natural sources is antimicrobial peptides (AMPs).

Antimicrobial peptides (AMPs) are typically cationic and usually composed of less than 50 amino acids [6]. AMPs are considered promising therapeutic agents due to their broad antimicrobial spectrum and unique mode of action against bacteria [7,8]. Unlike conventional antibiotics, positively charged AMPs are believed to exhibit electrostatic interaction with negatively charged bacterial membranes. Lipopolysaccharide (LPS) and lipoteichoic acid (LTA) are the major cell membrane components appearing in Gram-negative and Gram-positive bacteria. Several widely accepted models explain the antimicrobial mechanism of AMPs, including the barrel-stave model, toroidal model, or detergent-like action (carpet model) [9,10]. Most AMPs exert antimicrobial mechanisms by disrupting or permeabilizing bacterial membranes [11]. Moreover, AMPs also show various intracellular mechanisms against bacteria, including inhibiting DNA, RNA, and protein synthesis.

However, Gram-negative organisms release Lipopolysaccharide (LPS; endotoxin), which has been identified as a significant inflammatory trigger, and an uncontrolled LPS response contributes to the risk of localized inflammation [12]. Several proinflammatory cytokines, such as tumor necrosis factor-α (TNF-α), interleukin-1 β (IL-1β), and nitric oxide (NO) play a significant role in mediating inflammation. Recent studies have shown that many peptides efficaciously suppress the production of LPS-induced NO and cellular cytokines [13].

Despite the early promise of AMPs as alternatives to antibiotics, many AMPs are not suitable for therapeutic applications due to their high manufacturing cost, ease of degradability, high hemolytic activities, and high cytotoxicity. Another significant barrier to AMPs as therapeutics is their sensitivity to physiological salts. To overcome these limitations, short AMPs isolated from natural sources have been identified as less toxic, with high stability and low production cost [14].

In the previous study, a novel antimicrobial peptide named YS12 was isolated from the traditional Korean food kimchi. Peptide YS12 from *Bacillus velezensis* CBSYS12 showed antimicrobial activity against different multi-drug resistant bacteria [15]. In many studies, it has been suggested that AMPs in combination with antibiotics offer rapid bactericidal effects and lower the chance of pathogens developing resistance [16,17]. In the current investigation, we aimed to show the synergistic effect of peptide YS12 with commercial antibiotics using combinational therapy. Next, we confirmed that peptide YS12 maintained antimicrobial activity in the presence of different physiological salts. Meanwhile, cytotoxicity towards mouse macrophage Raw 264.7 cell and hemolytic activities of peptide YS12 were also measured compared with the standard well-known antimicrobial compound melittin. The present work also explored the underlying mechanism of antimicrobial peptide YS12 on different bacteria. In the initial stage of peptide–microbial membrane interaction, peptide YS12 was found to bind with LPS. Thereafter, we assessed the inner membrane permeability of peptide YS12 using fluorescent dye PI and ONPG against bacterial strains. Confocal laser microscopy was used to observe the localization of the peptide YS12 on the bacterial cell membrane. Finally, the anti-inflammatory effect of peptide YS12 was also investigated on LPS-stimulated murine macrophage RAW264.7 cells in vitro.

## 2. Results and Discussion

### 2.1. MIC of Peptide YS12 and Antibiotics against Different Bacterial Strains

The infections caused by different microbes significantly increased over time [18]. Specifically, Gram-negative bacteria (*E. coli*, *P. aeruginosa*) and Gram-positive bacteria (*S. aureus)* are the most common bacterium causing various severe infectious diseases. Table 1, presents the Minimum Inhibitory Concentration (MIC) of peptide YS12 and antibiotics against different bacterial strains. The data indicated that peptide YS12 showed prominent antimicrobial activity at MIC values of (6–12 µg/mL) against the isolated Gram-negative strains. The bacterial strain *E. coli* KCTC 1923 had a lower MIC value of 12 µg/mL to ciprofloxacin and 24 µg/mL to oxacillin against *P. aeruginosa* KCTC 1637. However, erythromycin and oxacillin showed MIC values higher than 96 µg/mL against *P. aeruginosa* and *S. aureus* KCTC 1928 strains, respectively.

### 2.2. Synergism Effect of Peptide YS12 with Conventional Antibiotics

Combined antibiotic therapy is considered a promising approach to combat bacteria and increase the potency of antibiotics. Specifically, combining AMPs with antibiotics has drawn attention as it often shows a stronger killing effect against bacteria [19]. In the above study, we aimed to determine the synergistic effect between peptide YS12 and antibiotics, including oxacillin, ciprofloxacin, or erythromycin by calculating the fractional inhibitory concentration index (FICI) against *E. coli* KCTC 1923, *P. aeruginosa* KCTC 1637 and *S. aureus* KCTC 1928. In Table 2, a significant synergistic effect was obtained for YS12 and ciprofloxacin against *E. coli* KCTC 1923, which achieved a fractional inhibitory concentration index (FICI) of 0.157. Similarly, combining peptide YS12 with oxacillin or erythromycin exhibited a synergistic effect (FICI of 0.325 to 0.415) on *E. coli* and *S. aureus* strains. The partial synergism (FICI > 0.5) was observed when YS12 was combined with ciprofloxacin or erythromycin against *P. aeruginosa* and *S. aureus*, respectively. Moreover, peptide YS12 combined with erythromycin showed additive or partial synergism (FICI of 1.00) against *P. aeruginosa*. The overall finding of this study proved that the peptide YS12 exhibited a strong synergistic effect with conventional antibiotics against different pathogenic strains.

**Figure 1:**
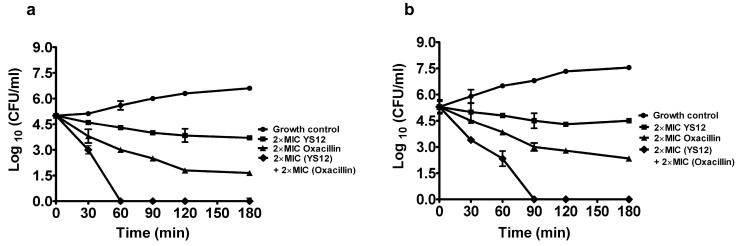
The synergistic effect of peptide YS12 with oxacillin against (**a**) *E. coli* KCTC 1923 (**b**) *P. aeruginosa* KCTC 1637. The time-killing effect of the combination of 2 × MIC (12 µg/mL) of peptide YS12 with 2 × MIC (96 µg/mL) oxacillin against *E. coli* and 2 × MIC (24 µg/mL) of peptide YS12 with 2 × MIC (48 µg/mL) oxacillin against *P. aeruginosa* was compared with the effect of 2 × MIC of peptide YS12, 2 × MIC of oxacillin alone, or untreated control.

After that, we observed the intense synergistic effect of peptide YS12 combined with the commercial antibiotic using the time-killing test. We evaluated the bactericidal effect of peptide YS12 with oxacillin against *E. coli,* and *P. aeruginosa* bacteria. Peptide YS12 and oxacillin showed a killing effect on both bacterial strains over 180 min. In Figure 1a, the data indicated that combining peptide YS12 with oxacillin at the concentration of 2 × MIC showed a rapid bactericidal effect. *E. coli* cells were completely killed within only 60 min. *P. aeruginosa* was slightly less sensitive compared to *E. coli.* In *P. aeruginosa*, the combination of peptide YS12 and oxacillin at 2 × MIC resulted in the reduction in the CFU from 5.3 logs to 2.4 logs at 60 min and destroyed the cell bacterial cells in 90 min (Figure 1b). These findings confirmed that the peptide YS12 with the antibiotic showed a faster killing effect than using the peptide YS12 or the antibiotic itself. Some studies also revealed the synergistic effect between AMPs and antibiotics [20]. The results suggested that combination therapy using AMPs and antibiotics may be an effective strategy to combat bacteria.

### 2.3. Stability Assay

A significant limitation of natural antimicrobial peptides (AMPs) is their possible degradation by different physiological concentrations of salts [21]. AMPs may exhibit a partial or even total loss of antimicrobial activity under different salt concentrations [22]. AMPs must maintain antimicrobial activities in salt-containing environments to be effective therapeutic agents. Numerous studies have suggested that monovalent Na^+,^ Mg^2+^, and Fe^3+^ may reduce the antimicrobial activity of naturally occurring AMPs [23]. Therefore, we determined the antimicrobial activity of peptide YS12 and melittin using different physiological salt concentrations. As shown in Table 3, the peptide YS12 had a minimal effect on Na^+^, Mg^2+^, and Fe^3+^ cations against bacterial strains. The peptide YS12 maintained antimicrobial activity (MIC~2 µg/mL) against *E. coli* and *P. aeruginosa* in the presence of Na^+^ and Fe^3+^, respectively. Similarly, for *S. aureus*, YS12 retained its stability (MIC~2–4 µg/mL) at various salt concentrations. The peptide YS12 showed a comparatively higher MIC value (4–8 µg/mL) against *E. coli* and *P. aeruginosa* in the presence of Mg^2+^. According to our findings, the peptide YS12 proved to have a strong tolerance to different salts. These results are consistent with the outcomes of salt stability studies from various AMPs [24,25].

### 2.4. Cytotoxicity and Hemolytic Activity of YS12

**Figure 2:**
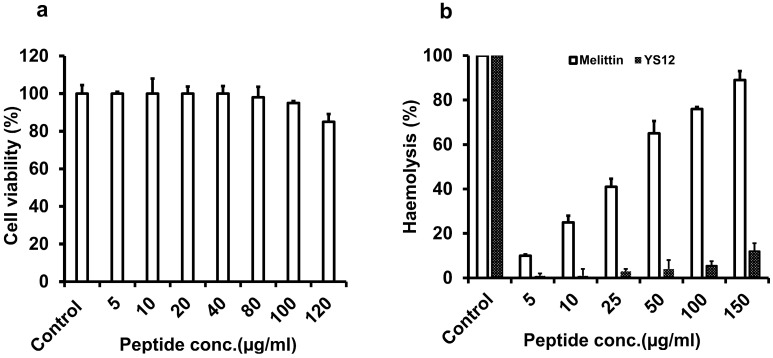
(**a**) Cell viability using Raw 264.7 cells was measured. Raw 264.7 cells were treated with peptide YS12 (5–120 µg/mL). (**b**) The hemolytic activity of peptide YS12 and Melittin with different concentrations (5–150 µg/mL) was determined. Each bar represents the mean ± SV obtained from three independent experiments, each performed in triplicate.

It has been assumed that AMPs exhibit considerable cell selectivity. Cytotoxicity and hemolytic activities are frequently determined to assess the cellular toxicity of AMPs [26]. To evaluate the cell selectivity more clearly, we checked the cytotoxicity of peptide YS12 on Raw 264.7 cells using the MTT reagent. According to the findings (Figure 2a), no cytotoxicity was observed up to 40 µg/mL in Raw 264.7 macrophage cells. Cell viability for YS12 was measured at approximately 83% at 120 µg/mL. Interestingly, the peptide YS12 caused great survival in Raw 264.7 cells. The hemolytic activity of peptide YS12 was also checked using red sheep blood. In Figure 2b, the peptide YS12 exhibited less than 15% hemolysis at the highest (150 µg/mL) concentration. In contrast, melittin was used as a negative control and demonstrated 76% hemolytic activity at 100 µg/mL. The low cytotoxic and hemolytic effects of AMPs were also reported in other studies [27,28].

### 2.5. The Potential Antimicrobial Peptide Mechanisms of Peptide YS12

#### 2.5.1. Liposome Preparation and Aggregation

**Figure 3:**
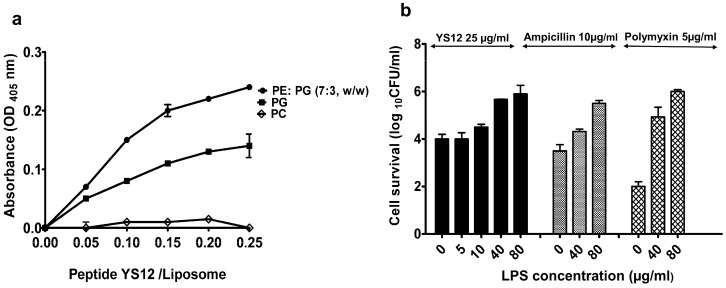
(**a**) Aggregation of large unilamellar vesicles (LUV). A quantity of 400 µM LUV with PE: PG (7:3, *w*/*w*) PG and PC were added to a solution containing different ratios of peptide YS12, and aggregation was observed at 405 nm. (**b**) *E. coli* cell survival was checked after treatment with lipopolysaccharide (LPS) either itself or in combination with ampicillin (10 µg/mL), polymyxin (5 µg/mL), and peptide (25 µg/mL).

LUVs are widely employed to mimic bacterial membranes [29]. The lipid bilayer of the microbial membrane is composed of phospholipids containing phosphoglycerol (PG), phosphoethanolamine (PE), phosphatidylcholine (PC), and cardiolipin that have negative charges. To investigate the interaction between peptide YS12 and bacterial membrane, we established different types of LUVs with PE: PG (7:3, *w*/*w*), PG, and PC, respectively [30]. The amount of aggregation caused by the peptide YS12 was measured using the turbidity of liposomes. PE: PG liposome suspension turbidity increased gradually and consistently at a peptide/lipid (P/L) ratio from 0.05 to 0.025. When the peptide/liposome ratio was 0.025, the turbidity was achieved at 0.025, as shown in Figure 3a. With increased peptide/liposome ratios, the turbidity of PG arises slowly compared with PE: PG. However, the peptide could not interrupt phosphatidylcholine (PC) liposomes. The stronger interactions of the peptide YS12 with bacterial membranes were confirmed through the aggregation of PE: PG and PG liposomes. AMPs were also shown to have an impact on bacterial membranes [31].

#### 2.5.2. LPS Binding Affinity of Peptide YS12

Gram-positive and Gram-negative bacterial membranes differ significantly in their molecular components and structure. The interaction between bacterial membranes and AMPs plays a vital role in microbial killing and developing new antimicrobial agents [32,33]. Typically, LPS acts as a barrier in Gram-negative organisms. We initially evaluated the binding affinity of peptide YS12 for bacterial endotoxin (LPS). The peptide YS12 could bind with LPS by electrostatic attraction to the *E. coli* surface. In Figure 3b, it was observed that LPS only slightly decreased the antibacterial activity of YS12 at lower concentrations of 5 µg/mL, but at 80 µg/mL, LPS successfully neutralized the bactericidal activity of YS12. Polymyxin B, an effective outer membrane binding agent, was employed as a positive control, and the bacteria was no longer sensitive at 80 µg/mL. Ampicillin as negative control also did not affect LPS. Based on the findings, YS12 seems to have a strong affinity for the LPS of Gram-negative bacteria.

**Figure 4:**
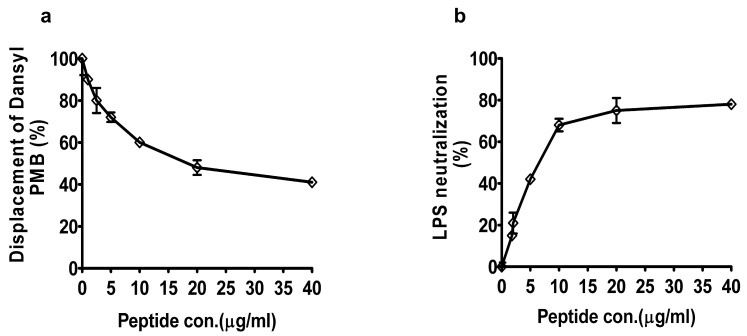
(**a**) Binding affinity of peptide for LPS (from *E. coli*) as analyzed using dansyl PMB. (**b**) LPS neutralization by peptide YS12 was determined using an endotoxin quantification kit.

We also conducted the dansyl-polymyxin B displacement experiment to quantify the LPS binding affinity of peptide YS12. DPX works effectively as an indicator for cationic bindings on both purified LPS and whole bacterial cells [34]. However, DPX is nonfluorescent in free solution but exhibits high fluorescence when bound with LPS [35]. As seen in Figure 4a, peptide YS12 was bound to LPS by replacing the dansyl PMB and decreased fluorescence from 90% to 40% at 2.5 and 40 µg/mL, respectively. The peptide YS12 exerted an electrostatic interaction with LPS that contributed to its excellent antimicrobial activity against Gram-negative bacteria. Additionally, we evaluated the LPS neutralization activity of peptide YS12 using a Limulus amebocyte lysate (LAL) test. Different concentrations of YS12 (2.5–40 µg/mL) and 1 ng/mL LPS were employed. In Figure 4b, we found that peptide YS12 neutralized LPS in a dose-dependent manner and showed 78% neutralization at the maximum concentration of 40 µg/mL. These results indicated that the peptide YS12 showed significant effects on LPS. Numerous studies have shown that AMPs have a higher affinity for binding LPS to Gram-negative bacteria [36].

#### 2.5.3. Peptide YS12 Damaged the Membrane Integrity of Bacteria

**Figure 5:**
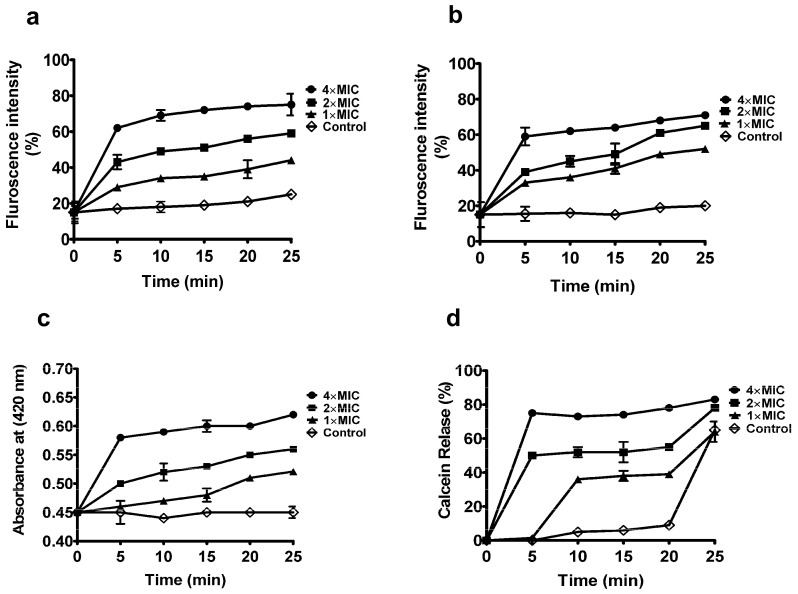
The potential antimicrobial mechanism on the bacterial membrane. PI uptake assay for membrane integrity of bacteria (**a**) *E. coli* ATCC 1923, (**b**) *S. aureus* KCTC 1928. (**c**) Release of cytoplasmic B-galactose of *E. coli* ATCC 1923 treated with peptide YS12 at different concentrations (1×, 2×, and 4 × MIC). (**d**) The calcein release rate treated with peptide YS12 was determined. 0.1% TritonX-100 was used as a positive control.

The positively charged AMPs have different mechanisms of action on bacteria, including disruption of the integrity of the cell membrane and leakage of intracellular material [37,38]. Initially, we confirmed that the peptide YS12 showed an excellent LPS binding affinity on the Gram-negative strain. In this study, we explored the effects of peptide YS12 on the inner membrane of bacterial strains (*E. coli*, *P. aeruginosa*) employing the fluorescent dye PI. Typically, the peptide treatment allowed PI to enter into bacterial cells [39]. In Figure 5a,b, it was shown that the presence of peptide YS12 at 1×, 2×, and 4 × MIC increased fluorescence intensity over time. The peptide YS12 exhibited the highest fluorescence of approximately 75% and 71% at 4 × MIC against *E. coli* and *S. aureus*, respectively. The result indicated that the peptide YS12 had an impact on the integrity of the bacterial cell membrane. 

Moreover, a colorimetric and spectrophotometric substrate (ONPG) is usually used to measure the β-galactosidase activity in the inner membrane. To assess the effect of peptide YS12 on the inner membrane of *E. coli*, ONPG was used as β-galactosidase. The bacterial cytoplasmic membrane consists of negatively charged hydroxyl phospho-lipids, which facilitate the binding of cationic AMPs [40]. In Figure 5c, peptide YS12 caused rapid permeabilization of the *E. coli* membrane. The fluorescence intensity was gradually increased in a time- and concentration-dependent manner. The experimental results using PI and ONPG demonstrated that the peptide YS12 affected the integrity of the bacterial inner membrane, which ultimately led to cell death. Furthermore, by measuring the release of calcein from negatively charged liposomes, the membrane permeabilization capability of peptide YS12 was also detected. Peptide YS12 resulted in membrane disruption by inducing over 80% calcein leakage from calcein-loaded liposomes at the highest concentration of 4 × MIC (Figure 5d). Collectively, our results revealed that the peptide YS12 damaged the bacterial membrane by increasing PI intake, cytoplasmic-galactosidase release, and calcein release. These results would provide a better understanding of the mechanism of action of antimicrobial peptides (AMPs). Other studies also suggested that the antimicrobial peptides (AMPs) treatment enhanced bacterial membrane destruction [41,42].

#### 2.5.4. Confocal Microscopy

**Figure 6:**
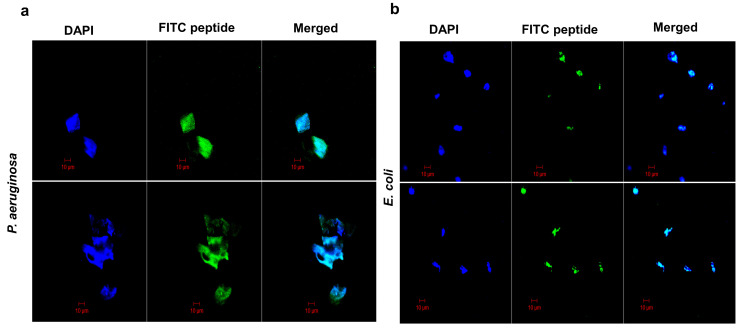
Localization of FITC-labeled peptide YS12 in bacterial cell membrane. (**a**) *P. aeruginosa* KCTC 1637 (**b**) *E. coli*. KCTC 1923 cells were incubated with peptide YS12 at the concentration of 4 × MIC. The bacterial cells were washed, fixed, and stained with DAPI (blue).

In this study, we employed Fluorescein isothiocyanate (FITC)-labeled YS12 dye to investigate the entrance of the peptide YS12 into bacterial cells. Peptide localization on bacterial cells was visualized using confocal laser microscopy. FITC-labeled YS12 corresponding to a 4 × MIC or 24 µg/mL and 4 × MIC or 48 µg/mL value was applied on *P. aeruginosa* KCTC 1637 and *E. coli* KCTC 1923 strains, respectively, for 30 min. In Figure 6a,b, we observed that FITC-labeled YS12 translocated into the bacterial cell by aggregating in the cytoplasm. The results of this study proved that peptide YS12 accumulated in the cytoplasm of the cells.

### 2.6. The Ability of Peptide YS12 to Inhibit Pro-Inflammatory Mediators in RAW 264.7 Cells

**Figure 7:**
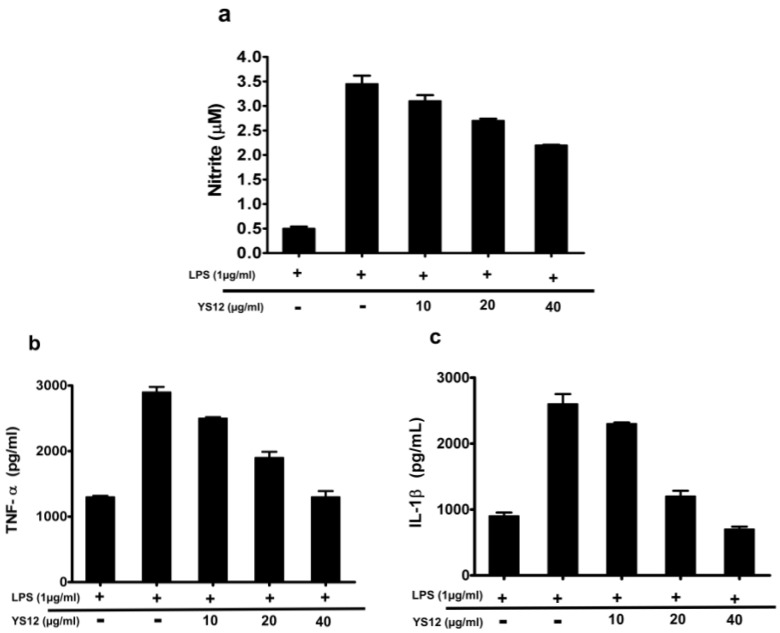
Effects of peptide YS12 on inhibiting proinflammatory cytokines including TNF-α, IL-1β, and NO. (**a**) The amount of NO was measured by Griess reagent in a concentration-dependent manner. (**b**) The effect of peptide YS12 was determined on cytokines including TNF-α, and (**c**) IL-1β. Each bar represents the mean ± SV of three independent trials.

AMPs possess remarkable anti-inflammatory effects by neutralizing LPS [43]. In bacterial infections, proinflammatory cytokines such as TNF-α and IL-1β are generated more readily, and excessive cytokines can induce organ damage and inflammation. We evaluated the effect of peptide YS12 on the secretion of LPS-stimulated cytokines (TNF-α, IL-1β) and NO in Raw 264.67 cells. The cells were treated with LPS (1 µg/mL) and peptide YS12 at a concentration of 10–40 µg/mL. The ELISA kit was used to measure TNF-α release. In Figure 7a, peptide YS12 showed a considerable effect on NO production in a concentration-dependent manner. In Figure 7b,c, we observed that peptide YS12 led to a significant reduction in the release of pro-inflammatory mediators, including TNF-α and IL-1β. Taken together, these results suggest that peptide YS12 may be an effective approach for developing a new anti-inflammatory agent.

## 3. Materials and Method

### 3.1. Materials and Reagents

Materials were obtained in the following manner: the strain CBSYS12 was isolated from the Korean traditional food kimchi; and PG (L-alpha-phosphatidyl glycerol), (PE) phosphatidylethanolamine, and PC (l-alpha-phosphatidylcholine) were acquired from Avanti polar Lipid (Alabaster, AL, USA). The following reagents were purchased from Sigma-Aldrich (St. Louis, MO, USA): LPS (from *E. coli*), MTT (3-(4,5-dimethylthiazol-2-yl)-2,5-diphenyl tetrazolium bromide), Polymyxin B, and propidium iodide (PI). From commercial sources, other chemical reagents of analytical grade were obtained.

### 3.2. Peptide YS12 Production and Purification

The production and purification of the antimicrobial peptide YS12 derived from *Bacillus velezensis* CBSYS12 was described in our previous paper [15].

### 3.3. Antimicrobial Activity Determination in Terms of MIC

The concentration that suppressed bacterial growth was identified using a minimum inhibitory concentration (MIC) assay described in our recently published paper [15].

### 3.4. Synergistic Effect of the Peptide with Different Antibiotics

The synergistic effects of peptide YS12 with other conventional antibiotics were investigated using the two-dimensional checkerboard method [44]. Peptide YS12 was added with serially diluted antibiotics into 96-well plates. Then, bacterial cells (approximately 10^6^ microorganisms) were mixed into each plate, and growth-control wells were filled with only the medium. OD_600_ was measured by a spectrophotometer after 18 h of incubation at 37 °C. The fractional inhibitory concentration (FIC) index was obtained using the following equation:

FIC index = FICA + FICB

[(MIC of drug A in combination/MIC of drug A alone) + (MIC of drug B in combination/MIC of drug B alone)]

FIC index was represented as follows: FICI ≤ 0.5 indicated synergy and 0.5 < FICI ≤ 1 denoted partial synergism.

### 3.5. The Time-Dependent Killing of the Synergistic Group

Combination therapy against the Gram-negative strains *E. coli* and *P. aeruginosa* was used to analyze the synergistic effect of peptide YS12 with the commercial antibiotic oxacillin [45]. Peptide YS12 at the concentration of 2 × MIC was mixed with the 2 × MIC of oxacillin. At different time intervals (30, 60, 90, 120, 150, and 180 min), the mixture was collected and put onto MHB agar plates. The bactericidal effect was determined by colony-forming units (CFUs). The experiment was also carried out without using antibiotics as the control.

### 3.6. Stability Assay

The stability assay was performed to determine the antimicrobial activity of peptide YS12 against different physiological salts following the previous article [46]. In Mueller Hinton Broth media, *E. coli* KCTC 1923, *P. aeruginosa* KCTC 1637, and *S. aureus* KCTC 1928 strains were grown and diluted to 2 × 10^5^ CFU/mL. The bacterial suspensions were incubated with peptide YS12 supplemented with different physiological salts, such as NaCl (100 mM, 150 mM), MgCl_2_ (0.5 mM, 1 mM), and FeCl_3_ (2 µM, 4 µM). To determine MIC, the experiment was carried out according to the method previously explained [15].

### 3.7. In Vitro Cytotoxicity Assay

The cytotoxicity of peptide YS12 was measured using 3-(4,5-dimethylthiazol-2-yl)-2,5-diphenyl tetrazolium bromide (MTT) reagent in Raw 264.7 cells [47]. In brief, Raw 264.7 cells were cultivated in a 96-well plate. The cells were incubated at 37 °C with 5% CO_2_ overnight. The cells were treated with increasing concentrations (5–120 µg/mL) of peptide for 24 h at 37 °C under 5% CO_2_ atmosphere. MTT was added to the well the next day at a 0.5 mg/mL concentration. The absorbance was monitored at 570 nm using an ELISHA reader (Molecular Devices, Sunnyvale, CA, USA). At least two replicates were given to each measurement.

### 3.8. Hemolytic Activity of YS12

The hemolytic activity of peptide YS12 using fresh red sheep blood was measured as previously reported with slight alteration [48]. Fresh red sheep blood cells were washed with PBS, and the supernatants were removed by centrifugation at 1000× *g* for 5 min. The peptide YS12 at 5–150 µg/mL was added to 96 well plates. Melittin was used as a control. A quantity of 4% RBC was put into each well and incubated at 37 °C. At 1000× *g*, the plate was again centrifuged for 5 min at 4 °C, and the supernatants were placed on a fresh 96-well plate. The optical density (OD) at a wavelength of 414 nm was measured using a microplate reader (Bio-Tek Instruments, Winooski, VT, USA). The absorbance of PBS-treated cells indicated zero hemolysis, while those treated with 0.1% Triton X-100 indicated complete hemolysis.

The hemolysis percentage was calculated using the formula shown below:

Hemolysis (%) = (Abs_sample_ − Abs_PBS_)/(Abs_triton_ − Abs_PBS_) × 100

### 3.9. The Potential Antimicrobial Mechanisms Study of Peptide YS12

#### 3.9.1. Preparation and Aggregation of Liposomes

Large unilamellar vesicles (LUVs) were developed using the freeze-thaw technique [49]. The liposome mixture was prepared by dissolving PE: PG (7:3, *w*/*w*), PG, and PC in chloroform. The dispersed liposomes were then removed with argon gas. The lipid film was dried, and then the solution was vortexed in PBS with a pH of 7.2 until a milky appearance developed in the suspension. LUVs were created utilizing freeze-thaw cycles under a water bath and liquid nitrogen. A polycarbonate membrane with a thickness of 0.2 m was used to extrude the suspensions. The peptide YS12 was added at 5, 10, 20, and 40 µg/mL to 400 µM LUVs composed of PE: PG, PG, and PC. Using a microplate reader, the enhanced absorbance was determined at 405 nm.

#### 3.9.2. Effect of Lipopolysaccharides (LPS)

The mechanism of action of AMPs is greatly dependent on their binding capability with the cell membrane [50]. The antibacterial activity of peptide YS12 against *E. coli* KCTC 1923 in the presence of LPS was identified. *E. coli* cells (approximately 6.0 × 10^7^ CFU/mL) were grown and diluted with tryptic soy broth. The cell suspension was mixed with lipopolysaccharides from *E. coli* O111: B at a concentration of 5, 10, 40, or 80 µg/mL. Peptide YS12 was added to a final concentration (25 µg/mL) against *E. coli.* Ampicillin at 10 µg/mL and Polymyxin at 5 µg/mL were used as positive and negative controls, respectively. The mixtures were incubated at 37 °C for 60 min. The stayer cells were measured by spreading tryptic soy agar.

#### 3.9.3. LPS Binding Assay Using Dansyl-Polymyxin B

A Danyl-polymyxin B displacement experiment was also carried out to determine the binding affinity of peptide YS12 for LPS [51]. LPS from *E. coli* and Danyl-polymyxin B were treated with pH 7.2 HEPES buffer (1 mL; 5 mM) to achieve optimal fluorescence. The peptide YS12 was added to the cuvette, and a spectrometer was used to measure the decrease in fluorescence. Excitation and emission wavelengths of the fluorescence were detected at 340 nm and 485 nm, respectively.

#### 3.9.4. Neutralization of LPS by Peptide

A chromogenic limulus amebocyte lysate assay was used to measure the LPS neutralization [52]. The peptide YS12 (5–40 µg/mL) and constant concentration of LPS (from *E. coli*) (1 ng/mL) were put in the pyrogenic sterile microtiter plate and incubated at 37 °C. The solutions were mixed and treated with equivalent amounts of Limulus amebocyte lysate reagent for 10 min. Following the addition of a chromogenic substrate solution, a yellow color appeared. An amount of 25% acetic acid was added to terminate the reaction, and the absorbance was measured at 580 nm.

#### 3.9.5. PI Uptake Assay

Membrane permeabilization assay of peptide YS12 was performed using fluorescent dye propidium iodide (PI) [53]. Briefly stated, *E. coli* and *S. aureus* bacteria were grown in MHB medium to the mid-log phase and diluted in sodium phosphate buffer (10 mM) to an OD_600_ value of 0.25. On a dark 96-well plate, each bacterium was treated with PI (final concentration, 20 µM). After mixing, peptide YS12 at 1×, 2×, and 4 × MIC was added to each well. The fluorescence spectrophotometer was used to measure the absorbance at 580 nm and 620 nm for the excitation and emission, respectively, and the percentage (%) of fluorescence intensity obtained from the peptide YS12 was represented relative to untreated controls.

#### 3.9.6. Inner Membrane Permeability Activity

Ortho-nitrophenyl-galactose (ONPG) was used to analyze the inner membrane permeabilization of the peptide YS12 against *E. coli* bacteria [54]. Bacterial cells were cultivated, collected, and resuspended in PBS (pH 7.2) to a final OD_420_ of 1.2. The peptide (1×, 2×, 4 × MIC) and ONPG (final concentration 1.5 mM) were placed on 96 well plates. The microplate reader was then used to measure the absorbance at 420 nm.

#### 3.9.7. Calcein Dye Leakage Assay

Calcein-loaded liposomes were prepared to imitate bacterial membranes. The liposomes were formed according to the method described in the earlier report [55]. Peptide YS12 at 1 × MIC, 2 × MIC, and 4 × MIC was loaded into liposomes (1:1, *v*/*v*). The fluorescence intensity (excitation 490 nm, emission 530 nm) was recorded at 0, 5, 10, 15, 20, and 25 min. The calcein release rate was calculated by the following equation:

The extent of calcein output was calculated as

(F_t_ − F_0_)/(F_max_ − F_0_),

where F_t_ represents the fluorescence of a peptide liposome/calcein solution at a particular time, and F_0_ and F_max_ indicate the initial fluorescence. F_t_ is the fluorescence of a peptide liposome/calcein solution at the time, and F_0_ and F_max_ are the initial fluorescence and the fluorescence following total liposomal disruption by the detergent (Triton X-100, 10% *w*/*w*), respectively.

#### 3.9.8. Confocal Microscopy

Gram-negative strains *E. coli* and *P. aeruginosa* were grown in LB broth to the mid-logarithmic phase. The cell suspensions were centrifuged and washed several times with a 10 mM PBS buffer (pH 7.5). The cells were treated with fluorescein isothiocyanate (FITC) labeled peptide YS12 (4 × MIC) at 37 °C. After an hour of incubation, the cells were centrifuged, pipetted into PBS buffer (pH 7.5), and then allowed to stand on a glass slide. Cell images with FITC-labeled peptides were observed using confocal laser microscopy (LSM 510, Carl Zeiss, Jena, Germany).

### 3.10. Inhibitory Effect of Peptide YS12 on the Production of Proinflammatory Mediators in LPS-Stimulated Raw 264.7 Cells

The anti-inflammatory effect of peptide YS12 was also investigated on LPS-stimulated murine macrophage RAW264.7 cells [56]. RAW264.7 (1 × 10^5^) cells were seeded in a 96-well culture plate overnight in triplicate. Cells were exposed to peptide samples (10, 20, and 40 µg/mL) and then stimulated with 1µg/mL of LPS purified from *E. coli* 0111:B4 (Sigma-Aldrich, St. Louis, MO, USA) for 16 h. After incubation, equal volumes of supernatant fractions and Griess reagent (1% sulfanilamide, 0.1% naphthyl ethylenediamine dihydrochloride, and 2% phosphoric acid) were mixed and incubated for 10 min. After incubation, the fluorescence was measured. Moreover, the inhibitory effect of peptide YS12 on the release of inflammatory cytokines such as TNF-α, and IL-1β was determined by ELISA. Raw 264.7 cells were incubated with peptide YS12 at 10–40 µg/mL and LPS (1 µg/mL) for 24 h. After incubation, the supernatants were measured for TNF-α, and IL-1β by ELISA.

### 3.11. Statistical Analysis

The average and standard deviation (SD) of the experimental data were represented by three replications per inspection. Graph Pad Prism software, version 5, was used to complete the statistical investigation.

## 4. Conclusions

In our previous study, we evaluated the antimicrobial activity of peptide YS12 against both Gram-positive and Gram-negative strains. In our present work, peptide YS12 showed a synergistic effect with commercial antibiotics. Enhanced salt-resistant properties and low cytotoxicity of peptide YS12 were also observed. In terms of mode of action, peptide YS12 exhibited direct antimicrobial activity by disrupting the integrity of the bacterial cell membrane. Additionally, peptide YS12 exerted potent anti-inflammatory activities. Based on the overall findings, we assume that peptide YS12 may be used as a promising candidate for treating infectious illnesses caused by drug-resistant strains and inflammation-related disorders.

## Figures and Tables

**Table 1:** The MIC of peptide YS12 and antibiotics against bacterial strains.

Microorganisms	Peptide YS12 and Antibiotics	MIC (µg/mL)
	YS12	6
	Oxacillin	48
*Escherichia coli* KCTC 1923	Ciprofloxacin	12
	Erythromycin	48
	YS12	12
	Oxacillin	24
*Pseudomonas aeruginosa* KCTC 1637	Ciprofloxacin	48
	Erythromycin	>96
	YS12	48
	Oxacillin	>96
*Staphylococcus aureus* KCTC 1928	Ciprofloxacin	96
	Erythromycin	24

**Table 2:** The FIC index of peptide YS12 and antibiotics.

Bacterial Strains	Peptide-YS12 Oxacillin		Peptide-YS12 Ciprofloxacin		Peptide-YS12 Erythromycin	
	ΣFIC	Interpretation	ΣFIC	Interpretation	ΣFIC	Interpretation
Gram-negative						
*E. coli* KCTC 1923	0.325	Synergism	0.157	Synergism	0.451	Synergism
*P. aeruginosa* KCTC 1637	0.501	Synergism	0.749	Partial synergism	1.00	Partial synergism
Gram-positive						
*S. aureus* KCTC 1928	0.931	Partial synergism	0.75	Partial synergism	0.415	Synergism

FIC of <0.5 was interpreted as synergism and 0.5 < 1.0 as an additive or partial synergism.

**Table 3:** Antimicrobial activity of peptide YS12 in the presence of different physiological salts.

	NaCl (mM)			MgCl_2_ (mM)		FeCl_3_ (µM)	
	Control 100		150	0.5	1	2	4
*E. coli* KCTC 1923							
peptide YS12	2	2	2	8	2	2	2
Melittin	4	4	2	4	4	2	2
*P. aeruginosa* KCTC 1637							
Peptide YS12	2	2	2	4	2	2	2
Melittin	2	2	2	2	2	4	4
*S. aureus* ATCC 1928							
Peptide YS12	4	2	2	4	2	2	2
Melittin	4	2	4	4	2	4	2

## Data Availability

The data presented in this study are available on request from the corresponding author.
